# Surgical Management of Adult Spinal Deformity Patients with Osteoporosis

**DOI:** 10.3390/jcm13237173

**Published:** 2024-11-26

**Authors:** Makeen Baroudi, Mohammad Daher, Krish Maheshwari, Manjot Singh, Joseph E. Nassar, Christopher L. McDonald, Bassel G. Diebo, Alan H. Daniels

**Affiliations:** Department of Orthopedic Surgery, The Warren Alpert Medical School, Brown University, Providence, RI 02912, USA; makeenb91@gmail.com (M.B.);

**Keywords:** osteoporosis, adult spinal deformity, proximal junctional kyphosis

## Abstract

Adult spinal deformity (ASD) commonly affects older adults, with up to 68% prevalence in those over 60, and is often complicated by osteoporosis, which reduces bone mineral density (BMD) and increases surgical risks. Osteoporotic patients undergoing ASD surgery face higher risks of complications like hardware failure, pseudoarthrosis, and proximal junctional kyphosis (PJK). Medical management with antiresorptive medications (e.g., bisphosphonates, SERMs, and denosumab) and anabolic agents (e.g., teriparatide, abaloparatide, and romosozumab) can improve BMD and reduce complications. While bisphosphonates reduce fracture risk, teriparatide and newer agents like romosozumab show promise in increasing bone density and improving fusion rates. Surgical adaptations such as consideration of age-adjusted alignment, fusion level selection, cement augmentation, and the use of expandable screws or tethers enhance surgical outcomes in osteoporotic patients. Specifically, expandable screws and cement augmentation have been shown to improve fixation stability. However, further research is needed to evaluate the effectiveness of these treatments, specifically in osteoporotic ASD patients.

## 1. Introduction

Adult spinal deformity (ASD) is a heterogeneous group of disorders that cause substantial disability in patients [1]. ASD is common and can occur in up to 68% of adults over 60 years of age [2]. Historically, conservative management was often favored in frail and osteoporotic patients due to the complex underlying pathology as well as high rates of postoperative complications such as potential catastrophic neurologic injury [3]. However, despite the limitations in medical management of this condition [4], improved understanding of disease mechanisms, advances in surgical techniques, ability to manage postoperative complications, and improved patient reported outcomes, operative intervention is becoming increasingly performed, with the latter showing better outcomes [5,6].

Osteoporosis is a condition of reduced bone mineral density (BMD) of at least 2.5 standard deviations below the mean hip mass (T-score) as defined by the World Health Organization [7]. Nearly 10 million Americans over the age of 50 are diagnosed with osteoporosis, with an estimated 34 million at risk [8,9]. Indeed, in a sample of 399 ASD patients undergoing long-segment spinal fusion, the prevalence of osteoporosis is ‘substantially’ higher than that observed in the general population (34.4% vs. 10.3%) [10]. Patients at risk of osteoporosis, such as women over 65 and post-menopausal women, are encouraged to undergo routine screening, although many ASD patients without risk factors may also have osteoporosis [11]. The most common screening tool is dual-energy X-ray absorptiometry (DEXA) scan [12]. If this is unavailable, a computed tomography (CT) scan can be used to measure Hounsfield units (HUs) of the lumbar spine [13]. HUs measurements at the planned upper instrumented vertebra (UIV) may be useful at predicting post-operative proximal junctional kyphosis/proximal junctional failure (PJK/PJF). In a retrospective cohort study of 63 ASD patients, Yao et al. noted an inverse correlation between HUs at the UIV/UIV + 1 and PJK rates [14]. This conclusion is mirrored by multiple studies where authors encourage using pre-operative HUs to assist with ASD surgery planning [15,16]. Chanbour et al. went further and identified that 163 HU may serve as a preliminary threshold at the UIV, mitigating the risk of mechanical complications in ASD patients [17].

Many research papers available discuss the management of osteoporosis; fewer ones look at managing osteoporosis in ASD patients. We aim to examine up-to-date evidence of medical management of osteoporosis in ASD patients prior to surgery and explore evidence-based intraoperative techniques to decrease complications for the vulnerable spine in these patients.

## 2. Methods

We conducted a comprehensive search of electronic databases, including PubMed, Google Scholar, and Embase, for relevant studies published between 2000 and 2024. We were keen to use the most up-to-date evidence, especially for papers on surgical techniques. Search terms included ‘adult spinal deformity’, ‘osteoporosis’, ‘spinal instrumentation’, ‘screw loosening’, ‘cement augmentation’, and ‘surgical outcomes’. Examples of exclusion criteria are spine surgery studies on pediatric patients, articles not written in English, and we avoided using abstracts where the ‘Results’ Section was not available.

## 3. Osteoporosis and Its Surgical Challenges

Within ASD surgery, osteoporosis poses a surgical challenge as a result of its impact on bone integrity [18,19]. The weakened vertebrae can compromise the stability of the spine, leading to an increased risk of postoperative complications such as instrumentation fixation failure, vertebral fractures, and subsequent spinal cord compression [20,21]. Adjacent segment vertebral fractures, leading to both sagittal and coronal malalignment, are especially problematic [22]. Correcting sagittal malalignment in osteoporotic patients requires careful consideration of bone quality, spinal alignment, and biomechanical stability [23]. This may explain why osteoporotic patients exhibit a higher than usual rate of instrument failure during surgical procedures [24]. The compromised bone makes it challenging to achieve adequate fixation and stability with standard surgical implants and techniques [25]. At the same time, the underlying density loss in osteoporosis can distort the microarchitecture of bone [26]. The increased osteoclast-mediated bone resorption coupled with the compensatory osteoblastic bone formation disrupts the natural healing process and impedes the fusion of bone segments after spine surgery [27].

## 4. Post-Surgical Outcomes

Osteoporosis is also associated with the risk of non-instrumentation surgical complications in ASD patients [10,28,29,30]. Most commonly, osteoporosis has been shown to increase the risk of intraoperative blood loss and postoperative DVT [30,31]. Despite the already high occurrence of short-term complications in this population, they are at an even greater risk of long-term complications and revision surgery compared to non-osteoporotic patients. In fact, a retrospective cohort study on 2564 ASD patients found that osteoporosis is one of the top predictors of revision surgery 2 years following surgery [32]. Another study of 2293 patients similarly demonstrated that osteoporosis is a significant risk factor for revision surgery at 5 years after surgery [33]. Notably, both studies found that using bone morphogenetic protein plays a protective role against revision surgery. Additionally, using a combined anterior–posterior approach instead of a posterior-only approach may also contribute to a lower reoperation rate [34]. A different retrospective study of 1044 patients added that patients with either osteopenia or osteoporosis had increased odds of revision, suggesting that even mild BMD loss may contribute to higher complication rates [35].

Likewise, Gupta et al. followed 399 patients undergoing ASD surgery in a retrospective study and demonstrated that 40.5% of patients with osteoporosis underwent revision surgery, compared to 28% of non-osteoporosis patients, and noted that osteoporotic patients had an increased risk of hardware failure, pseudoarthrosis, PJF, and infection [36]. Khalid et al. also found a twofold increased risk of PJK and distal junctional kyphosis [35]. More recently, a systematic review of 16 studies, encompassing 133,086 patients, found that the rates of PJK/PJF, screw loosening, and revision surgery after ASD surgery were all higher in patients with osteoporosis/osteopenia [37]. A 2024 meta-analysis of 28 studies, likewise, identified a greater risk of screw loosening and PJK following ASD surgery in osteoporotic patients [38].

## 5. Medical Management of Osteoporosis in ASD Patients

Appropriate pharmacologic management of osteoporosis has been shown to improve outcomes and prevent complications following ASD surgery [10,39]. See ‘Table 1’ for a summary of the different medications available.

### 5.1. Antiresorptive Medications

#### 5.1.1. Bisphosphonates (e.g., Alendronate, Pamidronate, Zoledronic Acid)

Bisphosphonates work by modulating bone mineralization through binding to hydroxyapatite crystals, thus leading to the inhibition of calcification [40]. Traditionally, bisphosphonates have been the most commonly used medication to treat osteoporosis [41]. However, in a large cohort study of 1040 osteoporotic/osteopenic patients undergoing fusion surgery, pre-operative bisphosphonates did not seem to have an effect on fusion rates [42]. In addition, a meta-analysis of seven studies that included 401 patients found that while bisphosphonates following spinal fusion surgery do not improve bone formation or fusion rates, they do reduce the frequency of vertebral compression fractures, loosening of pedicle screws, and cage subsidence [43]. However, a 2016 trial of 79 osteoporotic patients undergoing spinal fusion surgery found that prophylactic treatment of zoledronic acid (Reclast™/Zometra™) can improve fusion rate [44].

#### 5.1.2. Selective Estrogen Receptor Modulators (SERMs)

Tamoxifen (Nolvadex™, Soltamox™) is a first-generation SERM that has historically shown an ability to increase BMD in post-menopausal women through the upregulation of bone estrogen receptors (ERs) [45,46]. Second and third generation SERMs have shown similar ability, making them important options for treating osteoporosis and preventing fractures in postmenopausal women [47,48]. In terms of specific effect on the spine, the seminal MORE multicenter randomized controlled trial (RCT) of 7705 patients showed that raloxifene can increase BMD of the spine and reduce the risk of vertebral fractures [49]. However, typical adverse events, such as venous thromboembolism and breast cancer, still need to be considered when administering SERMs. Pre-clinical studies show aspects of using SERMs for better fusion rates and protection against adjacent segmental degeneration [50,51]. While these studies are promising, the efficacy of SERMs in osteoporotic patients undergoing ASD surgery requires further research.

#### 5.1.3. Denosumab (Prolia™, Xgeva™)

Denosumab is a human monoclonal antibody that increases bone density and decreases bone resorption through the prevention of binding of receptor activator of nuclear factor kippa-B ligand (RANKL) to its RANK receptor [52]. It was found to strengthen pedicle screw fixation and BMD in postmenopausal women [53]. An RCT of 76 osteopenic patients who underwent spinal fusion found that denosumab improved BMD of the lumbar spine and reduced bone turnover markers, while at the same time providing restoration of functional status [54]. Nevertheless, trials are needed to investigate the use of denosumab in ASD patients.

### 5.2. Anabolic Medications

#### 5.2.1. Teriparatide (Forteo™) and Abaloparatide (Tymlos™)

The first anabolic agent used to treat osteoporosis was teriparatide (TP), a human recombinant parathyroid hormone (PTH) [55]. On a cellular level, it acts on the PTH-1 receptor to induce a mitogenic effect on osteoblastic cells and reduce their apoptosis, thus leading to a higher number of bone-forming cells [56,57]. This improves osteoporosis of the spine by increasing BMD [58,59]. However, improvements in BMD with teriparatide rapidly decline following its cessation; thus, combination therapy with an antiresorptive agent is recommended to prevent this reversal [58,59,60,61,62]. Specifically, it seems that combining with raloxifene is more favorable for increased BMD than alendronate [63]. Furthermore, a 2018 randomized prospective study of 16 osteoporotic patients found that combination therapy of denosumab and TP can achieve better fusion rates than monotherapies [64].

Neoadjuvant TP treatment in low-BMD ASD patients is proposed to reduce postoperative complications [65]. In a prospective case series of 76 ASD surgery patients, 43 of them received prophylactic TP [66]. The treatment group had improved BMD and reduced incidence of PJK. In a prospective cohort study that compared pre-operative treatment of TP vs. low-dose bisphosphonates in 58 osteoporotic patients undergoing ASD surgery, TP was found to be more effective at preventing post-operative adjacent vertebral fractures, implant failure, and fusion failure [67]. Although using TP tends to show better fusion rates than bisphosphonates following spine surgery, this difference is often not clinically significant [68]. A retrospective study on 57 ASD patients found similar results; additionally, patients receiving TP had improved HU values by 20.8% compared to the non-TP patients [69]. Finally, the use of TP as PJF prophylaxis has been implicated recently. A 2024 prospective trial by Kim et al. on 63 osteoporotic patients undergoing ASD surgery showed a better ability for TP at preventing PJF than denosumab [70]. This potential prophylactic role of TP is supported by a 2023 meta-analysis of 13 studies, covering 745 spinal fusion patients, that showed TP’s ability to ‘significantly’ increase rates of fusion [71].

Abaloparatide (AP) is a synthetic parathyroid hormone-related peptide analog (PTHrP) that induces its anabolic effect through binding to the PTHR1 receptor [72]. It has shown efficacy at increasing BMD in the spine and at reducing vertebral body fractures [39,72]. This is especially the case in post-menopausal women [73,74]. It is a relatively novel drug; thus, less evidence exists on its usefulness in ASD patients compared to TP [75]. A common strategy is to provide osteoanabolic agents for 3–6 months pre-operatively and ongoing postoperatively for osteoporotic patients undergoing ASD surgery.

#### 5.2.2. Romosozumab (Evinity™)

Sclerostin is a protein that binds to osteoblasts via low-density lipoprotein receptors and inhibits the Wnt signaling pathway, which contributes to skeletal development and bone remodeling [76]. Romosozumab, a humanized monoclonal antibody, acts as a sclerostin inhibitor, thus reducing bone resorption and promoting osteoblast activity by preventing the inhibition of the Wnt signaling pathway [77]. It has shown its ability to increase vertebral BMD in postmenopausal women at a rate better than both TP or bisphosphonates [78,79,80]. It has also shown its ability to reduce the risk of vertebral fractures [81]. The efficacy of this medication on osteoporotic patients undergoing spine surgery is yet to be studied to the same extent as other medications [75].

## 6. Surgical Adaptations for the Osteoporotic Spine 

### 6.1. Age-Adjusted Alignment

Adjusting sagittal alignment targets may help in preventing future osteoporosis-related risks [82]. In a retrospective cohort study of 313 osteoporotic patients, sagittal alignment was found to be a strong predictor of osteoporotic-related fractures [83]. Depending on their age, ASD patients with severe sagittal deformity may have compensatory lower-limb extension to support their posture; thus, there is a need for age-adjusted sagittal alignment targets [84]. Additionally, it is important to avoid over-/under-correcting malalignment in ASD patients. A systemic literature review of six articles that studied age-adjusted alignment thresholds on 1825 ASD patients found that PJK was more common in patients with overcorrected sagittal deformity [85]. This finding was mirrored in a 2022 study on 409 ASD patients [86] (Table 2).

### 6.2. Fusion Levels

It seems that upper thoracic (UT) fusion is the more desirable option for osteoporotic ASD patients. Multiple studies investigated the choice of UIV selection and length of fusion in ASD patients. In a retrospective analysis of 875 patients, UT UIV was compared to lower thoracic (LT) UIV for long fusions [87]. Daniels et al. followed 303 ASD patients and found that while UT UIV had longer operation times and greater volume of intraoperative blood loss, it resulted in fewer postoperative PJK rates and in greater vertical sagittal axis improvement [88]. Therefore, if long operation time or significant blood loss are of concern, then LT fusion may be more appropriate; if alignment issues such as thoracic coronal plane deformity or PJK/PJF are present—which is the case in osteoporotic patients—UT fusion may be the favored approach [89].

The use of longer fusion constructs acts to increase the amount of fixation throughout the spine, thereby distributing stress away from individual vertebrae and decreasing the risk of fracture and screw pull-out [90]. Although postoperative complications can still occur, this risk appears to be lower when extending fusion length compared to shorter-segment fusions [91]. One retrospective study of 253 patients undergoing multi-level fusion with pelvic fixation, for instance, demonstrated a 4.3% failure rate of pelvic fixation, which is significantly lower than what has been suggested in prior literature [92]. On the other hand, in a retrospective study of 53 corrective fusion patients, fusion constructs ending at S1 were associated with a higher risk of L5/S1 instability, pseudoarthrosis, and other complications compared to constructs ending slightly higher up at the ilium [93]. However, in a more up-to-date 2021 retrospective cohort study on 349 ASD patients who underwent spinal fusion surgery, the 6-week postoperative sagittal alignment restoration was better in patients whose fusion extended to S1 compared to L4/L5, despite having greater PJK rates and angles [94]. Patient age can be the key to synthesizing such contradicting findings into a practical conclusion [95]. In their sample of 40 ASD surgical fusion patients, Taneichi et al. recommend stopping long fusion at L5 for patients ≥ 50 years old due to its favored aspect of causing ‘less severe disability’ [95]. However, such a decision should be considered carefully since 50% of their patients whose fusion stopped at L5 required additional fusion due to insufficient deformity correction.

### 6.3. Screws, Hooks, and Tethers

A study on 13 human cadavers found that using a combination of pedicle screws and laminar hooks showed greater instrumentation stiffness compared to using screws alone [96]. In a study on 191 ASD surgery patients, tethering (polyethylene-tether only or tether with crosslink) was found to significantly lower the rates of post-operative PJK [97]. In a 2023 review of clinical evidence for using tethers to prevent PJK in ASD surgery, 14 out of 15 studies agreed that tethering shows a reduction in PJK PJF rates [98]. Strap stabilization and band placement that can act similarly to tethers may be used but require more evidence into their efficacy [99,100]. However, none of these papers specify if these ASD patients were osteoporotic; thus, focused research is needed to determine the efficacy of tethering in osteoporotic patients.

### 6.4. Expandable Screws

While relatively new, expandable titanium screws have shown potential promise in spine surgery to increase fixation. These screws used on four osteoporotic human cadavers demonstrated better stability than standard titanium screws, requiring a greater level of force to cause implant failure [101,102]. They allowed for expansion within the vertebral body, maximizing contact area and improving anchorage strength [101,103]. This finding was also demonstrated in a study on 157 osteoporotic patients, where expandable screws showed the dual benefit of better fusion rates and a lower incidence of loosening [104]. This same finding has been supported by other human trials as well [105,106].

### 6.5. Cement Augmentation

Cement augmentation can augment the stability of the fixation construct and reinforce the weakened bone [107,108]. Polymethylmethymethacrylate-injected (PMMA) screws showed 149% improvement in pullout strength [109]. This technique not only improves the pullout strength of screws but also helps to redistribute stress and minimize the risk of adjacent vertebral fractures [110]. This has the potential to be even more helpful in ASD patients whose vertebrae are more prone to fractures and instability. Results from a study of 38 patients with osteoporotic spines found higher fusion rates in patients where PMMA-injected screws were used [111]. Biodegradable calcium phosphate can be used instead of PMMA where needed, as it has shown similar efficacy on a sample of 40 thoracolumbar vertebrae [110]. Pre-clinical data suggest that screws with pre-filled cement may offer better fixation results than injecting cement [112]. This is especially true if the screws are pre-filled with high-viscosity cement [113].

Vertebroplasty and kyphoplasty can stabilize fractured vertebrae, restore vertebral height and alignment, and reduce the pain associated with vertebral compression fractures [114,115,116]. In a pre-clinical study on 18 cadavers, Kebaish et al. found that prophylactic vertebroplasty at UIV level and above can reduce junctional fractures following long spinal instrumentation [117]. Additionally, in a case series on 41 patients, they found that this procedure can reduce the incidence of PJK/PJF following ASD surgery [118]. However, more research is needed here, as this reduction does not seem to carry on in the long term [119]. A meta-analysis of six RCTs, encompassing 877 patients, found that cementation and vertebroplasty result in improved pain relief in osteoporotic patients with vertebral compression fractures (VCFs) when compared to conservative management [120]. Similarly, in a retrospective study of 87 patients with 145 VCFs, balloon kyphoplasty demonstrated significant pain reduction [121]. A similar conclusion was reached in a RCT of 300 patients [122]. However, both techniques have been correlated with an increased risk of adjacent level fractures as well as cement leakage [121,123,124]. It must be noted though that when vertebroplasty and kyphoplasty are utilized together in an appropriate patient sample, there may be a decreased rate of adjacent level fracture [125].

### 6.6. Minimally Invasive Surgery

Intraoperatively, key decisions are made regarding the best surgical approach in these patients. There is a difference in opinion between orthopedic surgeons on when to use open vs. percutaneous approaches for osteoporotic patients undergoing ASD surgery. In general, extra visibility and accessibility of open procedures provide the ideal control that is often needed for correcting severe deformities and significant sagittal imbalance [126,127]. However, many orthopedic surgeons argue that, ideally, osteoporotic patients should not be instrumented due to the possibility of increased blood loss, infection, longer recovery, and implant failure [75,127]. Therefore, when fears of such complications are prominent, minimally invasive percutaneous techniques can be used in osteoporotic patients [128,129]. Several studies have shown better pain relief and a lower rate of postoperative complications for percutaneous surgery in osteoporotic patients [129,130]. However, the limited visibility in this technique risks screw misplacement [127]. Additionally, there may be a lack of sufficient stabilization in cases of severe osteoporosis [128,131].

### 6.7. Optimal Surgical Approach: Instrumentation vs. Conservative Management

The debate on whether to perform spinal instrumentation on osteoporotic patients with ASD revolves around balancing the high risk of complications against the potential benefits of surgery. Critics who argue against instrumentation cite that the fragile nature of osteoporotic bone leads to a higher rate of complications such as screw loosening, pseudarthrosis, implant failure, and adjacent vertebral fractures [132,133]. In practical terms, this could mean delayed recovery and diminished functional improvements [134]. Additionally, there is an increased risk of blood loss and infection [128,130]. Therefore, conservative management, including bracing, physical therapy, and pharmacological treatments, is often recommended as an initial approach to avoid such risks [135].

However, instrumentation may provide substantial benefits, including improved alignment and pain relief in patients with severe deformities or debilitating pain, where conservative measures are more likely to fail [136]. Recent advancements in surgical techniques, such as cement-augmented pedicle screws and expandable screws, have demonstrated improved fixation and reduced hardware-related failures in osteoporotic patients [137].

New minimally invasive techniques also offer faster recovery times and fewer complications [23]. Moreover, several studies have shown better pain relief and a lower rate of postoperative complications for percutaneous surgery in osteoporotic patients [129,130]. However, cases of severe deformities and significant sagittal imbalance may require open surgery as it offers more accessibility [129,130]. Crucial elements to the success of instrumentation in osteoporotic patients are adequate pre-operative assessment and patient selection. Optimizing BMD via appropriate pharmacological treatment can improve bone quality and reduce the likelihood of postoperative complications [138]. These measures, combined with improved surgical techniques, can help mitigate the risks associated with operating on osteoporotic bone [128,129]. Therefore, the decision to instrument should be made on a case-by-case basis, weighing the patient’s deformity severity, overall health, and the experience of the treating physician.

## 7. Clinical Cases

### 7.1. Case #1

#### 7.1.1. History

**Figure 1:**
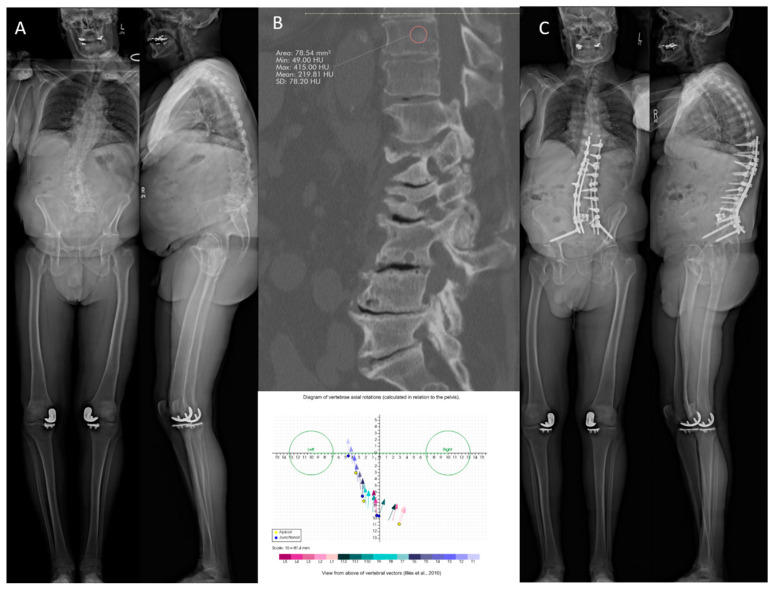
(**A**) Baseline anteroposterior and lateral standing radiographs. (**B**) Hounsfield units at the UIV on sagittal CT scan, mean HU 219.8 (**top**), and axial plane vertebral translation (**bottom**). (**C**) Anteroposterior and lateral standing radiographs 2 years post-operatively.

A 70-year-old osteoporotic male patient presented with a kyphoscoliosis deformity with severe underlying spinal stenosis at L3–L4 and L4–L5, and progressive debilitating back pain along with neurogenic claudication and a functional right foot drop (Figure 1). The patient had a T score of −1.0 in the left hip, 2.9 in the lumbar spine, and −3.7 in the left forearm. In addition, T10 had 220 HU, which is higher than 163, and had minimal axial plane translation, leading to its selection as the planned UIV [17]. The patient did not receive medical optimization for osteoporosis due to insurance denial and adequate HU of the proposed UIV. The patient underwent an L5–S1 anterior lumbar interbody fusion (ALIF) with a T10 to pelvis posterior decompression, fusion, and multiple osteotomies. Anchors and tethers were placed at T9 for PJK prophylaxis.

#### 7.1.2. Follow-Up

Two years post-operatively, the patient has no complaints. The standing X-rays demonstrate appropriate postoperative alignment with no evidence of implant subsidence, complications, evidence of PJK, or hardware failure (Figure 1).

### 7.2. Case #2

#### 7.2.1. History

**Figure 2:**
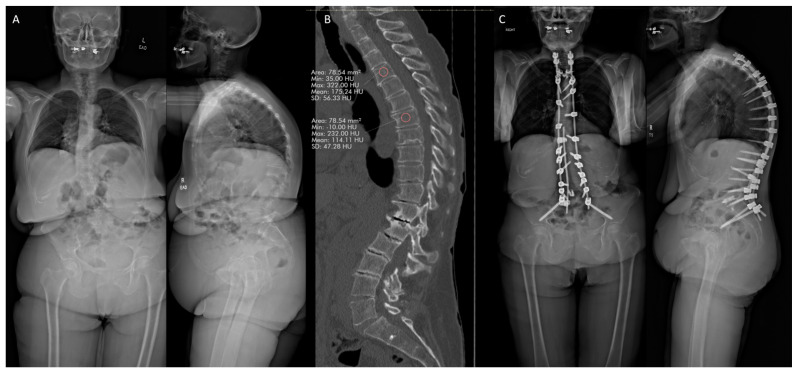
(**A**) Baseline anteroposterior and lateral standing radiographs. (**B**) Hounsfield units at the UIV on sagittal CT scan, mean HU 175.2. (**C**) Anteroposterior and lateral standing radiographs 2 years post-operatively.

A 63-year-old osteoporotic female patient presented with severe thoracolumbar spinal deformity, with ongoing severe axial back pain and significantly limited ability to stand and walk (Figure 2). The patient had a T score of −2.5 in the left hip and −2.2 in the lumbar spine. In addition, T4 had 175 HU, which is higher than 163, leading to its selection as the planned UIV [17]. The patient did not receive medical optimization for osteoporosis due to patient refusal to undergo medical management and adequate HU of proposed UIV. The patient underwent a T4–pelvis posterior decompression and fusion with multiple posterior column osteotomies. Anchors were placed at T3, and tethers were placed at T5 for PJK prophylaxis.

#### 7.2.2. Follow-Up

Two years post-operatively, the patient has no complaints. The standing X-rays demonstrate appropriate postoperative alignment with no evidence of implant subsidence, complication, evidence of PJK, or hardware failure (Figure 2).

### 7.3. Case #3

#### 7.3.1. History

**Figure 3:**
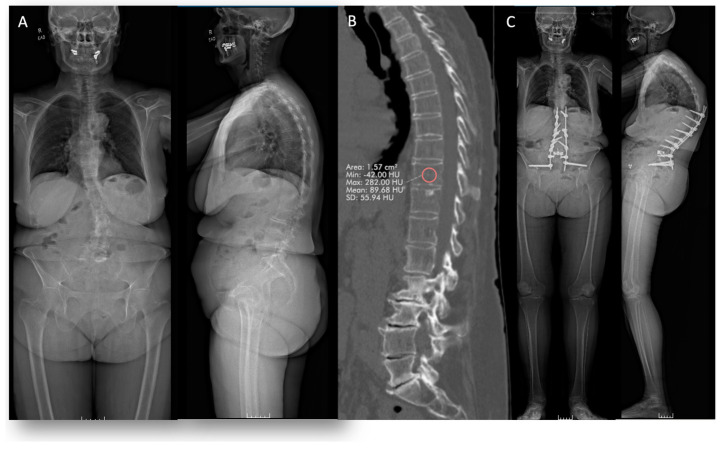
(**A**) Baseline anteroposterior and lateral standing radiographs. (**B**) Hounsfield units at the UIV on sagittal CT scan, mean HU 89.7. (**C**) Anteroposterior and lateral standing radiographs 2 years post-operatively showing proximal junctional kyphosis (PJK).

A 74-year-old osteopenic female patient presented with multilevel severe central and foraminal stenosis and thoracolumbar spinal deformity, with back pain and limited ability to ambulate (Figure 3). The patient had a T score of −1.7 in the lumbar spine. In addition, T10 had 89.7 HU, which is lower than 163, making it not the most optimal UIV in terms of bone mineral density [17]. The patient underwent a T11–pelvis posterior decompression and fusion with custom longitudinal rods and bilateral S2AI pelvis screws. Ligament augmentation was performed at UIV, UIV + 1, and UIV + 2 to minimize the risk of PJK.

#### 7.3.2. Follow-Up

Two years post-operatively, the patient comes back with complaints of back pain. The standing X-rays show PJK (Figure 3).

## 8. Conclusions

Osteoporosis presents a unique surgical challenge for ASD patients due to weakened vertebrae, altered bone remodeling, and increased risk of sagittal malalignment. These issues can lead to increased rates of revision surgery and instrument failure in osteoporotic patients. Medical management has varied levels of evidence. Anti-resorptive options such as bisphosphonates have reasonable evidence to support their use in osteoporotic ASD patients, whereas estrogen receptor modulators and denosumab require more human trials to establish efficacy in ASD. Similarly, anabolic options such as teriparatide are currently better supported by evidence than romosozumab. Surgical adaptations may overcome the challenges posed by osteoporotic patients in ASD surgery. Techniques such as adjusted alignment targets, higher and longer fusion constructs, cement augmentation of screws and/or the UIV, use of tethers and hooks, and potentially expandable screws have shown efficacy in osteoporosis and other conditions that cause vulnerable vertebrae. Further high-quality research is necessary to determine the effect of these interventions on outcome measures and to determine the true efficacy of some of the newer technologies discussed.

## 9. Limitations and Future Directions

The literature reviewed for this research article relied for the most part on clinical data. Studies that tested an intervention on animal subjects were not used to evaluate the efficacy of such an intervention. The authors were keen to include the most up-to-date evidence available for each intervention. Moreover, perhaps, the varying strength and abundance of clinical evidence for certain surgical techniques and medications poses a challenge to this review’s ability to confidently compare one intervention to another. Another limitation posed by the available literature is that patient follow-up periods in most clinical trials were within two years, making subsequent findings less generalizable for the long term.

As future research emerges in this field, studies should focus on obtaining more robust pharmacological evidence for osteoporotic patients specifically undergoing ASD surgery. Ideally, such evidence would come from prospective double-blinded RCTs, as many papers on this topic are retrospective in nature.

## Figures and Tables

**Table 1:** The advantages and disadvantages of using different medications for optimizing osteoporosis management in ASD surgical patients [40,41,42,43,44,45,46,47,48,49,50,51,52,53,54,55,56,57,58,59,60,61,62,63,64,65,66,67,68,69,70,71,72,73,74,75,76,77,78,79,80,81].

Medication	Mechanism of Action	Advantages	Disadvantages	Critical Appraisal of Evidence
Teriparatide (Forteo™, Eli Lilly and Company, Indianapolis, IN, USA)	Stimulates osteoblasts to increase BMD	Improved fusion rates and reduced PJK in ASD surgery. Better fusion rates when combined with denosumab.	Rapid decline in BMD following cessation.	Available studies suggest its use combined with other therapies. Larger long-term trials are required.
Abaloparatide (Tymlos™, Radius Health, Inc., Waltham, MA, USA)	Similar to teriparatide	Reduces vertebral fractures and improves BMD in osteoporotic patients.	Limited evidence is available on ASD patients.	Focused research is needed. Available evidence addresses osteoporosis patients in general, rather than ASD patients.
Romosozumab (Evinity™, Amgen Inc., Thousand Oaks, CA, USA)	Increases BMD via promoting bone formation and inhibiting resorption	It seems to be more effective than teriparatide and bisphosphonates. Reduces vertebral fractures.	Cardiovascular side effects.	RCTs demonstrate superior efficacy in increasing BMD in post-menopausal women. However, specific research on its use on ASD patients is needed.
Denosumab (Prolia™, Amgen Inc., Thousand Oaks, CA, USA)	Inhibits resorption by targeting RANKL	Improved fusion rates when used in combination with TP.	Rapid BMD loss when ceased. Rare adverse effects include osteonecrosis of the jaw (ONJ) and atypical femoral fractures.	Rapid bone loss following treatment cessation raises concerns over long-term viability.
Raloxifene (Evista™, Eli Lilly and Company, Indianapolis, IN, USA)	Increases BMD by acting as a SERM	Similar to denosumab.	Less effective than bisphosphonates in reducing fractures.	Weak evidence for standalone use to prevent fractures. Limited literature of use on ASD patients.
Alendronate (Fosamax™, Merck & Co., Inc., Rahway, NJ, USA)	Reduces bone resorption	Common use includes maintaining BMD following teriparatide therapy.	Prolonged use risks atypical femoral fractures and ONJ.	Extensive evidence backing its use for osteoporosis, especially when combined with other agents. Moreover, more evidence needs to prove similar efficacy in ASD patients. Observational studies suggest its ability to reduce compression fractures and cage subsidence.

RANKL: receptor activator of nuclear factor kappa-B ligand; ONJ: osteonecrosis of the jaw; SERM: selective estrogen receptor modulator.

**Table 2:** The advantages and disadvantages of different surgical techniques in ASD surgical patients with osteoporosis [82,83,84,85,86,87,88,89,90,91,92,93,94,95,96,97,98,99,100,101,102,103,104,105,106,107,108,109,110,111,112,113,114,115,116,117,118,119,120,121,122,123,124,125,126].

Surgical Technique	Advantages	Disadvantages	Critical Appraisal of Evidence
Age-adjusted Alignment	Helps prevent future osteoporosis-related fractures. Accounts for compensatory lower-limb extension in patients with severe sagittal deformity.	Potential risk of over- or under-correction, leading to PJK.	Most studies supporting its use are observational and retrospective cohort studies, which may limit generalizability and need further validation.
Fusion Levels (UT vs. LT Fusion)	UT fusion reduces PJK and improves sagittal alignment. Longer fusion constructs distribute stress away from individual vertebrae, reducing the risk of fractures and screw pullout.	UT fusion leads to longer operation times and increased intraoperative blood loss compared to LT fusion. Longer fusion constructs increase surgical complexity and potential for postoperative complications.	Retrospective studies favor UT fusion for ASD patients, particularly osteoporotic individuals. Although such findings are consistent across studies, the notably limited sample size, however, puts the strength of this evidence under scrutiny.
Screws, Hooks, and Tethers	Increases stiffness of the instrumentation. Tethering significantly reduces rates of PJK and PJF.	Limited evidence exists for the efficacy of tethering in osteoporotic ASD patients.	Stronger evidence is needed as most of the current evidence comes from cadaver and retrospective studies.
Expandable Screws	Greater stability and resistance to implant failure compared to standard screws. Improved fusion rates and reduced screw loosening in osteoporotic patients.	Clinical validation is needed for this relatively new technique.	Most results available come from early-stage studies. Long-term outcomes yet to be extensively studied.
Cement Augmentation (PMMA, Calcium Phosphate)	Significantly improves the pullout strength of screws. Redistributes stress and reduces the risk of adjacent vertebral fractures. Cementation techniques (vertebroplasty and kyphoplasty) reduce pain and stabilize fractured vertebrae.	Potential risks of adjacent level fractures and cement leakage.	More research is needed to confer long-term benefits, as pain relief seems to be short-lasting.

## Data Availability

Not applicable.
